# Supplementary material for: Nrf2 pathway activation promotes the expression of genes related to glutathione metabolism in alcohol-exposed astrocytes
Source: PeerJ. 2024 May 31;12:e17541. doi: 10.7717/peerj.17541 (PMC11146317; doi:10.7717/peerj.17541)
Supplement: Supplemental Information 8 [file peerj-12-17541-s008.docx]

**EXPERIMENTAL DESIGN**

**Definition of experimental and control groups**

CTX TNA2 astrocytes were divided into three groups: control, TBHQ (TBHQ + alcohol), and alcohol. To investigate the impact of TBHQ on astrocytes exposed to alcohol, the cells in the TBHQ group were treated with 40 µM TBHQ for 24 hours, following established protocols[8]. Subsequently, the medium was replaced with either normal complete medium (control group) or complete medium containing an alcohol concentration of 75 mM [9] (alcohol and TBHQ groups). After another 24-hour culture period, the cells were collected for subsequent experiments.

Number within each group

The biological replicates were 3 for each group.

**SAMPLE**

**Description**

CTX TNA2 astrocytes were inoculated with a density of 2×10^5^/ml and cultured with 6-well plates, and RNA was extracted after experimental intervention.

**Processing procedure**

If frozen - how and how quickly?

No frozen.

If fixed - with what, how quickly?

No fixed.

Sample storage conditions and duration (especially for FFPE samples)

Total RNA was extracted by TRIzol method immediately after the intervention.

**NUCLEIC ACID EXTRACTION**

**Procedure and/or instrumentation**

**Name of kit and details of any modifications**

RNA was obtained from CTX TNA2 astrocyte samples using TRIzol® reagent. Specific operations are as follows

​ After the culture solution was sucked out, 1ml of Trizol was added to each hole of the 6-well culture plate, and the solution of Trizol cells was blown up and sucked into a 1.5ml dynamic EP tube.

Absorb Trizol into 1.5ml dynamic EP tube (apply dynamic gun tip)

Let stand at room temperature for 5 minutes;

Add 0.3ml of chloroform, cover tightly and mix manually for 15 seconds;

2 - 3 minutes for the temperature;

Low temperature centrifuge 4℃, 12000g centrifuge for 15 minutes;

Approximately 0.4ml of the supernatant is transferred to a 1.5ml dynamic EP tube;

Add 0.5ml of isopropyl alcohol, mix well and let stand at room temperature for 10 minutes;

Low temperature centrifuges, 10,000 g centrifuges for 10 minutes;

Discard the supernatant, add 1ml 75% ethanol (prepared with DEPC treated water) and mix well;

Low temperature centrifuge 4℃, 7000g centrifuge for 5 minutes;

Discard the supernatant and dry at room temperature for 5 minutes;

DEPC treated water is dissolved in 20ul and stored at -80℃

The total RNA concentration of the sample was detected using an ultraviolet spectrophotometer;

**Details of DNase or RNAse treatment**

RNA was obtained from CTX TNA2 astrocyte samples using TRIzol® reagent and reverse-transcribed with the PrimeScript™ RT reagent Kit with gDNA Eraser (Perfect Real Time RR047A) (Takara). All experimental procedures were performed as specified in the kit instructions. The kit contains the gDNA Eraser, which removes genomic DNA in exactly 2 minutes. Among them, the reagent PrimeScript RT Enzyme Mix I has an RNase inhibitor.

**Contamination assessment (DNA or RNA)**

OD_260/280_ values of RNA samples were detected by ultraviolet spectrophotometer (Thermo), and OD_260/280_ values of all samples were greater than 1.8 and less than 2.

**Nucleic acid quantification**

**Instrument and method**

The RNA to be measured was dropped into the detection area, and the RNA concentration of the sample was detected by ultraviolet spectrophotometer.

**RNA integrity method/instrument**

RNA integrity was monitored by agarose gel electrophoresis, and the 28S/18S of each sample were between 1.7 and 2.1.

**Inhibition testing (Cq dilutions, spike or other)**

The target gene dissolution curves of all samples were unimodal.

**REVERSE TRANSCRIPTION**

**Complete reaction conditions**

**Amount of RNA and reaction volume**

Step 1

5×gDNA Eraser Buffer 2.0 μl

gDNA Eraser 1.0 μl

Total RNA 1.0 ng

RNase Free dH2O up to 10 μl

42℃ 2 min

4℃

Step 2

The reaction liquid of Step 1 10.0 μl

PrimeScript RT Enzyme Mix I 1.0 μl

RT Primer Mix 1.0 μl

5×PrimeScript Buffer 2 4.0 μl

RNase Free dH2O 4.0 μl

Total 20 μl

37℃ 15 min

85℃ 5 sec

4℃

**Priming oligonucleotide (if using GSP) and concentration**

RNA was reverse-transcribed with the PrimeScript™ RT reagent Kit with gDNA Eraser (Perfect Real Time RR047A) (Takara) in this experiment. The kit includes reagent PrimeScript Buffer, which contains dNTP Mixture.

**Reverse transcriptase and concentration**

RNA was reverse-transcribed with the PrimeScript™ RT reagent Kit with gDNA Eraser (Perfect Real Time RR047A) (Takara). The kit includes reagent PrimeScript RT Enzyme Mix I, which contains Reverse transcriptase and RNase Inhibitor.

**Temperature and time**

37℃ 15 min

85℃ 5 sec

4℃

**Manufacturer of reagents and catalogue numbers**

PrimeScript™ RT reagent Kit with gDNA Eraser (Perfect Real Time RR047A) (Takara)

**qPCR TARGET INFORMATION、PROTOCOL 、OLIGONUCLEOTIDES and VALIDATION**

The primer sequence used in this study is from the published article “Liver tumor promoting effect of etofenprox in rats and its possible mechanism of action”(PMID: 22467020), which has been validated and used. TB Green Premix Ex Taq II（Tli RNaseH Plus）(Code No. RR820A) was selected for this study. All experimental procedures were conducted as specified in the kit instructions.

**Probe sequence**

NM_001009920 glutathione S-transferase Yc2 subunit Gsta5 (Yc2)

Forward agaccaaagccattctcaactaca

Reverse ctccttcatgtccttcccataga

**DATA ANALYSIS**

**qPCR analysis program (source, version)**

Roche Group LightCycler96.

**Cq method determination**

Cq method=15.

**Outlier identification and disposition**

No outliers appear in this experiment.

**Results of NTCs**

No specific peak of NTC appeared.

**Justification of number and choice of reference genes**

The relative expression was calculated using the 2^−△△Ct^ method and estimated relative to GAPDH.

**Description of normalisation method**

All data were normalized by dividing by the mean of the control group before statistical analysis.

**Number and stage (RT or qPCR) of technical replicates**

​During the experimental sample addition process, each sample was repeated with 2 Wells.

**Repeatability (intra-assay variation)**

In this experiment, the difference of CT values between the two holes was less than 0.1.

**Statistical methods for result significance**

For qRT-PCR analysis, the differences among the three groups were evaluated using a one-way analysis of variance (ANOVA) followed by a post hoc test to determine the least significant difference. A significance level of P < 0.05 was considered statistically significant.

**Software (source, version)**

IBM SPSS Statistics 21
