# Supplementary material for: Nrf2 pathway activation promotes the expression of genes related to glutathione metabolism in alcohol-exposed astrocytes
Source: PeerJ. 2024 May 31;12:e17541. doi: 10.7717/peerj.17541 (PMC11146317; doi:10.7717/peerj.17541)
Supplement: Supplemental Information 9 [file peerj-12-17541-s009.docx]

Highlights

1. Alcohol exposure can increase the oxidation level in astrocytes, regulate the expression of glutathione metabolism pathway genes, and reduce the expression of the GSTA5 gene.

2. NRF2 agonist treatment suppressed oxidation in alcohol-treated astrocytes, regulated the expression of glutathione metabolism pathway genes, and promoted GSTA5 gene expression.

3. TBHQ could regulate ether lipid metabolism and reduce the contents of LysoPC(18:0) and PAF in astrocytes after alcohol exposure.
